# Retraction Note: Identification of proteins involved in transcription/translation (eEF 1A1) as an inhibitor of Bax induced apoptosis

**DOI:** 10.1007/s11033-025-10898-1

**Published:** 2025-08-08

**Authors:** Damilare D. Akintade, Bhabatosh Chaudhuri

**Affiliations:** 1 School of Life Sciences, Medical School, University of Nottingham, Nottingham, NG7 2UH UK; 2 Leicester School of Pharmacy, De Montfort University, Leicester, LE1 9BH UK

**Retraction Note: Molecular Biology Reports (2020) 47:6785–6792**

10.1007/s11033-020-05736-5

The Editor-in-Chief has retracted this article. After publication, it was brought to the attention of the publisher that two bands in Fig. 3G(c) appear to overlap, and that 3G(b) c-Myc Ab appears to overlap with β-actin bands in Fig. 9E of another article [1] by the same authors, under consideration at the same time. The authors have not provided a response to these concerns. The Editor has lost confidence in the data and conclusions of this article.

The Authors did not respond to correspondence from the Publisher about this retraction.

## References

[1] Damilare D, Akintade B (2020) The effect of copy number on α-synuclein’s toxicity and its protective role in Bax-induced apoptosis, in yeast. Biosci Rep 30 September 40(9):BSR20201912. 10.1042/BSR20201912 10.1042/BSR20201912 PMC7468099 32794578

